# Size-Segregated Particle Number Concentrations and Respiratory Emergency Room Visits in Beijing, China

**DOI:** 10.1289/ehp.1002203

**Published:** 2010-11-30

**Authors:** Arne Marian Leitte, Uwe Schlink, Olf Herbarth, Alfred Wiedensohler, Xiao-Chuan Pan, Min Hu, Matthia Richter, Birgit Wehner, Thomas Tuch, Zhijun Wu, Minjuan Yang, Liqun Liu, Susanne Breitner, Josef Cyrys, Annette Peters, H.-Erich Wichmann, Ulrich Franck

**Affiliations:** 1 Core Facility Studies, Helmholtz Centre for Environmental Research–UFZ, Leipzig, Germany; 2 University of Leipzig, Faculty of Medicine, Department of Environmental Medicine and Hygiene, Leipzig, Germany; 3 Physics Department, Leibniz Institute for Tropospheric Research, Leipzig, Germany; 4 Peking University, School of Public Health, Department of Occupational and Environmental Health, Beijing, People’s Republic of China; 5 State Key Joint Laboratory of Environmental Simulation and Pollution Control, College of Environmental Sciences and Engineering, Peking University, Beijing, People’s Republic of China; 6 Institute of Epidemiology, Helmholtz Zentrum München—German Research Center for Environmental Health, Neuherberg, Germany; 7 Environment Science Center, University of Augsburg, Augsburg, Germany; 8 Department of Epidemiology, Ludwig-Maximilians-University, Munich, Germany

**Keywords:** emergency room visits, particle number concentration, particle surface area concentration, particulate matter, short-term effects, time-series analyses, ultrafine particles

## Abstract

**Background:**

The link between concentrations of particulate matter (PM) and respiratory morbidity has been investigated in numerous studies.

**Objectives:**

The aim of this study was to analyze the role of different particle size fractions with respect to respiratory health in Beijing, China.

**Methods:**

Data on particle size distributions from 3 nm to 1 μm; PM_10_ (PM ≤ 10 μm), nitrogen dioxide (NO_2_), and sulfur dioxide concentrations; and meteorologic variables were collected daily from March 2004 to December 2006. Concurrently, daily counts of emergency room visits (ERV) for respiratory diseases were obtained from the Peking University Third Hospital. We estimated pollutant effects in single- and two-pollutant generalized additive models, controlling for meteorologic and other time-varying covariates. Time-delayed associations were estimated using polynomial distributed lag, cumulative effects, and single lag models.

**Results:**

Associations of respiratory ERV with NO_2_ concentrations and 100–1,000 nm particle number or surface area concentrations were of similar magnitude—that is, approximately 5% increase in respiratory ERV with an interquartile range increase in air pollution concentration. In general, particles < 50 nm were not positively associated with ERV, whereas particles 50–100 nm were adversely associated with respiratory ERV, both being fractions of ultrafine particles. Effect estimates from two-pollutant models were most consistent for NO_2_.

**Conclusions:**

Present levels of air pollution in Beijing were adversely associated with respiratory ERV. NO_2_ concentrations seemed to be a better surrogate for evaluating overall respiratory health effects of ambient air pollution than PM_10_ or particle number concentrations in Beijing.

There is consistent evidence that particulate matter (PM) with an aerodynamic diameter < 10 μm or 2.5 μm (PM_10_ or PM_2.5_, respectively) is adversely associated with respiratory morbidity and mortality (Alfaro-Moreno et al. 2007; Health Effects Institute 2003; Pope and Dockery 2006). Some findings suggest that associations are stronger for finer than for coarser particles (Kan et al. 2007; Peng et al. 2008; Wichmann et al. 2000) and that there are qualitative differences between the health effects of different particle size fractions (Samuelsen et al. 2009). Physicochemical parameters such as size, shape, distribution, number, and volume of airborne particles determine the potential to induce inflammatory injury, oxidative damage, and other biological effects, which are all stronger for smaller-size fractions of particles (Oberdörster et al. 2005; Valavanidis et al. 2008). Ultrafine particles (UFP; particles with a diameter < 100 nm) may contribute more than other particle size fractions to the observed health effects, because they dominate total particle number and surface area concentrations and have a high deposition efficiency in the pulmonary region (Delfino et al. 2005; Frampton 2001; Wichmann 2007).

Because of the limited availability of measurement data, few epidemiologic studies have investigated associations between particle number concentrations in different size ranges and daily respiratory morbidity or mortality, and their findings have been inconsistent. Wichmann et al. (2000) reported in a study conducted in Erfurt, Germany, with a population of approximately 200,000, that associations with total mortality were comparable for an interquartile range (IQR) increase in UFP [relative risk (RR) = 4.1%; 95% confidence interval (CI), 0.1–8.2] and PM_2.5_ (RR = 4.9%; 95% CI,1.1–8.8), whereas cause-specific mortality showed a somewhat stronger association with respiratory diseases compared with cardiovascular diseases and other causes. Peters et al. (1997) reported that inverse associations with peak expiratory flow (PEF) in asthmatic patients were stronger for an IQR increase in particle number concentrations of UFP than mass concentrations of fine particles (aerodynamic diameter 0.1–0.5 μm). Penttinen et al. (2001) reported that daily mean particle number concentrations, but not particle mass concentrations, were negatively associated with daily PEF deviations in adult asthmatics, whereas the strongest associations were found with particles in the ultrafine range. In a study conducted in Copenhagen, Denmark, Andersen et al. (2008) reported associations between pediatric asthma hospital admissions and IQR increases in accumulation-mode particles (0.1–1 μm), UFP (< 0.1 μm), and nitrogen oxide (NO_x_) concentrations.

On the other hand, associations between PM and hospital admissions due to cardiovascular and respiratory disease in the elderly in Copenhagen appeared to be mediated mainly by PM_10_ or accumulation mode particles rather than urban background UFP (Andersen et al. 2008). In addition, Osunsanya et al. (2001) found no association between UFP and respiratory symptoms or PEF in adult patients with chronic airflow obstruction in Aberdeen, Scotland.

Most studies of air pollution and health have been conducted in Western Europe or North America. Differences between Asian and Western populations in overall health status, lifestyle and age structure of the populations, and exposures to different air pollution mixtures and levels might influence associations between human health and air pollution. Studies conducted in Asian countries indicate that associations are similar to those in Western countries (Health Effects Institute 2004). Nevertheless, local authorities need population-specific information on current health risks of air pollution to develop air pollution control strategies adapted to local conditions.

The aim of this study was to analyze the role of different particle size fractions in the size range of 3 nm–1 μm with respect to respiratory health in Beijing, China.

## Data and Methods

The study was conducted from March 2004 to December 2006 (1,036 days) in Beijing, China.

### Data

**Figure 1:**
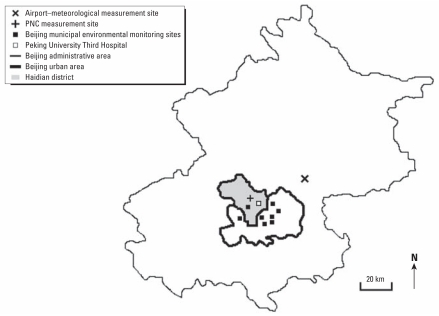
Map of Beijing, the study area, with administrative (outline) and urban (inner area) area, Haidian district (grey), and measurement stations.

Particle measurements were performed at a background measuring station on the campus of the Peking University (PKU) located in the northwestern part of Beijing in the Haidian district (Figure 1). The campus area is primarily residential and commercial, without heavy traffic or industrial sources. The inlet of the sampling system was placed 20 m above ground on top of a six-floor building located > 500 m away from major roads. Local emission sources within a 1-km radius include vehicular traffic and fuel combustion for domestic cooking, heating, and construction. A study of the spatial variability of PM_2.5_ mass and chemical composition in 1999–2002 showed only minor differences between a PKU campus site and an urban measurement site located approximately 10 km southeast from the PKU measurement site. The setup of the measurement station is described in detail elsewhere (e.g., Wehner et al. 2004, 2008).

We continuously measured aerosol number size distributions between 3 nm and 1 μm. Sampling was done using a Twin Differential Mobility Particle Sizer (TDMPS; Birmili et al. 1999), consisting of two Hauke-type differential mobility analyzers and two condensation particle counters (models 3010 and 3025; TSI Inc., St. Paul, MN, USA) which covered the size range from 3 nm to 800 nm (mobility diameter) and an Aerodynamic Particle Sizer (APS; model 3321; TSI Inc.) which measured particles between 800 nm and 1 μm (aerodynamic diameter). To combine both measurements, APS data were transformed from aerodynamic diameter to Stokes diameter assuming an effective particle density of 1.7 g/cm^3^ for the particles > 800 nm, because these particles are dominated by sulfate and crustal material in Beijing (Yao et al. 2003). A low-flow PM_10_ inlet was used to minimize contamination by large dust particles. The ambient aerosol was dried in a diffusion drier before entering the air-conditioned laboratory to avoid condensation of water in the inlet systems during warm and humid days in summertime. The data were corrected for losses due to diffusion and sedimentation within the inlet line. Size-dependent losses for the TDMPS inlet line were estimated using empirical particle loss corrections (diffusion and gravitation) from Willeke and Baron (1993). Losses of 4-nm and 10-nm particles were estimated to be 35% and approximately 10%, respectively.

Number size distributions were converted to particle number concentrations (PNC) and particle surface area concentrations (PSC) assuming spherical particles. For our analysis, we calculated daily means for 3–10 nm, 10–30 nm, 30–50 nm, 50–100 nm, 100–300 nm, and 300–1,000 nm size fractions. Total particle number or surface area concentration were computed as the sum of all fractions, and UFP as the sum of particle number concentrations < 100 nm.

In addition, daily particle mass (PM_10_), sulfur dioxide (SO_2_), and nitrogen dioxide (NO_2_) concentrations were gathered from the monitoring network of the Beijing Environmental Protection Bureau. Measurements were obtained as averages of eight fixed monitoring sites located in different parts of the urban area (Figure 1).

Daily mean meteorologic data from a measurement station near the Beijing Capital International Airport (Figure 1) were gathered from an internet weather service (Weather Underground 2009) because measurements from the meteorologic station closest to the particulate measurement station were incomplete. The Pearson correlation coefficients for valid days between the two data sources were 0.99 and 0.95 for daily air temperature and relative humidity, respectively, indicating a good agreement.

We collected data on hospital emergency room visits (ERV) from the Peking University Third Hospital, located in the Haidian district (Figure 1), where patients within 10 km of the measurement site were likely to be treated (personal communication with hospital doctors). The data acquisition system of the hospital recorded only patients who did not stay longer than 1 day. A standardized form was completed by medically trained study personnel at PKU, School of Public Health, by abstracting the data from the medical records. A database was built on the basis of these files, and respiratory clinical end points were coded according to the *International Classification of Diseases, 10th Revision* (ICD-10) [World Health Organization (WHO) 1993] for respiratory diseases (ICD-10 codes J00–J99). Total respiratory ERV comprise acute upper respiratory infections (J00–J06), pneumonia (J18), acute bronchitis (J20), other diseases of the upper respiratory tract (J30–J39), and chronic lower respiratory diseases (J40–J47). In addition, location of permanent residence was recorded for each case; based on this information, only patients from Haidian district were considered for further analysis. Because of an outbreak of the severe acute respiratory syndrome in China in 2003, all patients with body temperature > 38°C were separately treated in the hospital, and these cases were excluded. Assignment of the diagnoses to ICD-10 disease categories were quality-assured by a nosological expert from the Third Hospital, resulting in a good agreement, as the percentage of misclassification was about 4%.

### Statistical analysis

We applied a time-series analysis using generalized additive Poisson regression models (Hastie and Tibshirani 1987) to estimate associations between air pollutants and respiratory ERV.

In the first step, a base model without pollutants was built for identifying pollutant-independent variability. Long-term trends and seasonality were represented by a smooth function of calendar time, and a categorical variable for day of the week was forced into the model as hospital emergency room admissions showed weekly and yearly patterns. As further potential confounders, we considered the meteorologic parameters air temperature, air pressure, and relative humidity. We accounted for time-delayed effects of these variables using arithmetic means for days up to 7 days before the visit. Additional influences of the year, season (December–February, March–May, June–August, September–November), month, day of the month and holiday, represented by categorical variables were investigated as well. The base model was selected according to Akaike’s information criterion (Akaike 1974) and statistical significance of the covariates (*p* < 0.05). Nonlinear effects were modeled by regression splines with an automatic smoothness selection (Wood 2006). The smoothness of the trend function was manually adjusted based on the absolute value of the sum of the partial autocorrelation function (Touloumi et al. 2006).

The final base model consisted of the categorical variables holiday, weekend, and season, the linear influence of same-day relative air humidity, and the smooth functions of calendar time, air temperature, represented by the mean of the present and two previous days, and same-day air pressure.

The base model was extended by including air pollutants. Delayed effects up to a maximum of 5 days were estimated by polynomial distributed lag (PDL) models (Zanobetti et al. 2003), cumulative effects models that represented the delayed effect as a moving average, and single lag models that incorporated the delayed effect as a lagged variable into the model. We constrained the lag coefficients in PDL models to follow a third-degree polynomial of the lag number. Two-pollutant cumulative effects models included PM_10_ or NO_2_ in addition to another air pollution variable, with each modeled according to the same lag (e.g., same-day total particle number concentration and same-day NO_2_ concentration).

To explore the robustness of the models, we performed several sensitivity analyses for associations with particle number concentrations from 100 to 300 nm (PNC_100–300_) that varied the lag pattern of temperature, relative humidity, and air pressure, and used different degrees of smoothness for the time trend function. Additionally, for PDL models we varied the number of the maximum lag and the degree of the polynomial order.

All statistical analyses were done using the mgcv package in the R software (R Development Core Team 2003), version 2.90.

## Results

In total, we identified 15,981 cases of respiratory ERV in the study period, including 12,798 (80%) due to acute upper respiratory infections followed by 1,954 (12%) due to lower respiratory diseases. The daily mean of respiratory ERV was 15, with a minimum of 0 and a maximum of 88 patients per day (Table 1). Because of technical difficulties and maintenance, 218 days of particle size–segregated measurements were missing. Consequently, effect estimates for particulate air pollutants other than PM_10_ are based on 818 days of data (13,055 respiratory ERV were recorded in this period). Particle number concentrations were dominated by particles < 300 nm and particle surface area concentrations by particles in the range of 50–1,000 nm (Table 1). The time series of respiratory ERV showed a seasonal and weekly pattern, with more cases during the cold season and a peak on the weekend and on holidays (data not shown).

Size-segregated particle number and surface area concentrations were correlated with meteorologic and other air pollution variables [Table 2; see also Supplemental Material, Table 1 (doi:10.1289/ehp.1002203)]. Particle number concentrations of smaller particles (< 50 nm) and UFP were negatively correlated with air temperature, relative air humidity, and SO_2_. UFP and NO_2_ concentrations were not correlated when all days were included in the analysis (Table 2) but were moderately correlated when restricted to summer (*r* = 0.45), winter (*r* = −0.33), or transitional months (*r* = 0.16). There were small negative correlations between NO_2_ and PNC_3–10_ (−0.16) and PNC_10–30_ (−0.09) and a moderate positive correlation between NO_2_ and PNC_30–50_ (0.22) and PNC_50–100_ (*r* = 0.43). Particle number and surface area concentrations of larger particles (> 100 nm) as well as PSC_total_ were moderately correlated with relative air humidity, NO_2_, and PM_10_. PNC_total_ was moderately correlated with all meteorologic (air temperature, relative air humidity, and air pressure) and air pollutant (SO_2_, NO_2_, and PM_10_) variables. In addition, correlations between adjacent particle size classes were high.

Respiratory ERV increased by 5% [RR = 1.05 (95% CI, 1.02–1.08)] with an IQR increase (4,400 cm^−3^) in PNC_100–300_ with a 1-day lag (PDL model) [Table 3; see also Supplemental Material, Table 2 (doi:10.1289/ehp.1002203)]. Effect estimates for an IQR increase in NO_2_ were similar in magnitude to estimates for IQR increases in particle number and surface area concentrations of particles > 100 nm. Some inverse associations were observed with UFP and particle number concentrations of fractions < 50 nm. In most cases PNC_50–100_ was positively associated with respiratory ERV based on PDL models, although some single lag model associations were inverse.

Effect estimates for PNC, PSC, and NO_2_ were mostly higher in magnitude after adjustment for PM_10_ [Table 4; see also Supplemental Material, Table 3 (doi:10.1289/ehp.1002203)]. Associations with PNC and PSC were comparable in magnitude but less precise after adjustment for NO_2_, with borderline *p*-values (*p* < 0.09) only for PNC_100–300_ and PSC_100–300_ cumulative effects over 3 days (present and previous 2 days). In two-pollutant models, associations between respiratory ERV and NO_2_ were more consistent than those for other pollutants.

Associations with PNC_100–300_ were robust to variations in model parameters [see Supplemental Material, Table 4 (doi:10.1289/ehp.1002203)] in the sensitivity analyses.

## Discussion

We observed adverse associations between respiratory ERV and NO_2_ and particle number and surface area concentrations in several size ranges. Effects estimates for IQR increases in both particle number and surface area concentrations of 100–300 nm and 300–1,000 nm particles were comparable with estimated effects for IQR increases in NO_2_ concentrations, and effect estimates for NO_2_ were more consistent than those for other exposures in two-pollutant models. In most cases, particles < 50 nm were not positively associated with respiratory ERV, whereas particles in the size range of 50–100 nm were adversely associated with respiratory ERV, both being fractions of UFP.

Our findings are consistent with results from other studies reporting that levels of air pollution, often represented by PM_10_, PM_2.5_, NO_2_, SO_2_, O_3_, or UFP, are associated with short-term increases in ERV for respiratory complaints (Knol et al. 2009; Peel et al. 2005; Stieb et al. 2009; Tolbert et al. 2007).

Interestingly, we did not observe adverse associations of respiratory ERV with small particles (< 50 nm) but did observe positive associations with particle number concentration for particles > 100 nm. Andersen et al. (2008) investigated the association between short-term exposure to size-segregated particles and hospital admissions due to respiratory diseases in Copenhagen among the elderly (age > 65 years). They reported associations between total particle number concentrations and respiratory disease admissions (RR = 1.04; 95% CI, 1.00–1.07) comparable with the associations of PNC_100–300_ and PNC_300–1,000_ that we found.

Particle surface area concentrations also were adversely associated with total respiratory ERV, in agreement with Sager and Castranova (2009). This would be expected, as we derived particle surface area concentrations from particle number concentrations. Toxicologic studies report that surface area plays an important role in determining the biological activity of smaller particles, as they occupy less volume, resulting in a larger number of particles with a greater surface area per unit mass and an increased potential for biological interaction and absorption of chemical compounds (Oberdörster et al. 2005). However, it is not yet possible to measure particle surface area directly on a continuous scale.

Adverse associations between total respiratory ERV and both particle number and surface area concentrations remained after adjustment for particle mass (PM_10_). In two studies of respiratory health outcomes and air pollution, associations with particle number concentrations diminished after controlling for PM_2.5_ (Andersen et al. 2008; Halonen et al. 2008). Unfortunately, we did not have PM_2.5_ data available.

NO_2_ was associated with total respiratory ERV, in agreement with other studies (Peel et al. 2005; Stieb et al. 2009; Tolbert et al. 2007). NO_2_ itself has adverse health effects at high concentrations (> 200 μg/m^3^) (WHO 2005), but such levels are rare in Beijing. NO_2_ originates mainly from combustion processes and traffic, which are major sources of air pollution in Beijing (Sun et al. 2004), and NO_2_ is often correlated with other pollutants. Traffic-related air pollution has been associated with respiratory diseases in several studies (Brunekreef et al. 2009). In our two-pollutant models, associations with NO_2_ were most consistent. When controlling for NO_2_, associations with 100–300 nm stayed moderately significant (*p* < 0.09). A pollutant that exhibits a relatively strong association in a multipollutant model may be acting as a surrogate for an unmeasured or poorly measured pollutant (Peel et al. 2005). In addition, associations with pollutants based on single-pollutant models may be attributable to an association with another pollutant that is correlated with the measured pollutant. Typically, epidemiologic studies (including ours) cannot verify to what extent associations with NO_2_ are attributable to NO_2_ itself or to other pollutants correlated with NO_2_. Therefore, we cannot rule out the possibility that observed associations for NO_2_ were due to NO_2_-correlated air pollution.

We gathered daily concentrations of SO_2_, NO_2_, and PM_10_ from the Beijing Environmental Protection Bureau, but PM_2.5_ concentrations were not available. Because of size limitations, we did not investigate associations with disease-specific outcomes. Associations within population subgroups, such as the elderly or children, and disease specific analyses have suggested variation in effects and susceptibilities (Halonen et al. 2008). Associations between cardiovascular diseases and particle mass concentrations were not in the scope of our study, but are under investigation. Guo et al. (2009) reported that PM_2.5_, NO_2_, and SO_2_ concentrations were associated with hospital ERV for cardiovascular diseases in Beijing.

Exposure assessment and misclassification of exposure is a well-recognized limitation of epidemiologic time-series studies. Measurements from only one station were used for the particle size distribution data, and we were not able to assess spatial variation in particle number concentrations. Our measurement site may be considered as an urban background station because of its location (20 m height and > 500 m from a major road). Average particle number size distributions at a PKU measurement site and another regional measurement site, located approximately 50 km south of the PKU, were similar (Yue et al. 2009), confirming that our measurement site may be considered as an urban background station. In Beijing, concentrations of fine PM (PM_2.5_ and PM_10_) are evenly distributed over the urban area, including the Haidian district (Cheng et al. 2007; Wang et al. 2009). In urban areas, differences of absolute particle number concentrations between different measurement sites can be great (Krudysz et al. 2009), with the largest variations in ultrafine (< 100 nm) and coarse (2,500–10,000 nm) particles and more homogenous spatial distributions of accumulation mode particles (100–2,000 nm) (Monn 2001). The low correlation between NO_2_—as an urban average, representing traffic exhaust levels and the main source of UFP—and UFP might suggest low correlation between UFP at the measurement site and UFP levels elsewhere in the city. On the other hand, moderate correlations between UFP and NO_2_ were observed for subgroups, for example, seasons or particle subfractions of UFP. Personal levels of UFP can differ substantially, such as in proximity to traffic (Kaminsky et al. 2009). However, Buzorius et al. (1999), Cyrys et al. (2008), and Puustinen et al. (2007) showed that daily temporal correlations of particle number concentration time series between different monitoring sites are high. The authors concluded that using one carefully chosen monitoring site is a reasonable approach to characterize exposure of particle number concentrations in epidemiologic time-series studies. When associations are estimated by Poisson regression model in time-series studies, Lipfert and Wyzga (1995) argued that exposure misclassification will reduce the precision of effect estimates, resulting in wider CIs, but will not bias estimates. On the other hand, bias away from the null can occur in time-series studies of multiple pollutants, such as this one, when measurement errors of different pollutants are correlated (Zeger et al. 2000).

The air pollution mixture in Beijing is different from that of Western cities. The main sources of particulate air pollution in Beijing are coal burning, traffic, and dust from long-range transport (Sun et al. 2004). The mean particle number concentrations in urban areas in Europe or North America are 60–80% of the Beijing values, and particulate mass concentrations in Beijing are about three times higher than in Europe (Wehner et al. 2008). Additionally, differences between populations with respect to age, diet, sex, ethnicity, and state of health have to be taken into account when comparing results from Beijing with other cities in China or abroad.

Our findings should be interpreted cautiously, because exposure to particle number concentrations was based on data from a single background-monitoring site. In addition, the possibility that some effects might have occurred by chance cannot be excluded. To verify our findings with respect to different pollutants and particle fractions, further studies should be conducted, including analyzing the spatial correlation of pollutant time series.

## Conclusions

Present levels of air pollution were associated with respiratory ERV in Beijing, China. NO_2_ concentrations in Beijing appeared to be a better surrogate measure than PM_10_ or particle number concentrations for evaluating respiratory health effects of an air pollution mixture.

## Figures and Tables

**Table 1:** Descriptive statistics for daily mean values of respiratory ERV, meteorologic variables, and air pollution variables.

Variable	No. of valid days	Mean ± SD	Minimum	Maximum	IQR
Respiratory ERV	1,036	15 ± 10	0	88	12
Air temperature (°C)	1,036	13 ± 11	−10	31	18
Relative humidity (%)	1,036	58 ± 22	9	100	37
Air pressure (hPa)	1,036	1,017 ± 10	992	1,048	16
SO_2_ (μg/m^3^)	1,036	87 ± 88	6	600	100
NO_2_ (μg/m^3^)	1,036	63 ± 38	5	290	40
PM_10_ (μg/m^3^)	1,036	120 ± 83	10	570	90
UFP (1/cm^3^)	818	22,000 ± 9,800	5,600	76,000	11,000
PNC_total_ (1/cm^3^)	818	29,000 ± 10,000	7,400	87,000	12,600
PNC_3–10_ (1/cm^3^)	818	3,600 ± 4,400	85	41,000	3,880
PNC_10–30_ (1/cm^3^)	818	6,900 ± 3,800	1,200	30,000	4,300
PNC_30–50_ (1/cm^3^)	818	4,900 ± 1,800	890	14,000	2,300
PNC_50–100_ (1/cm^3^)	818	6,700 ± 2,800	630	19,000	3,600
PNC_100–300_ (1/cm^3^)	818	6,300 ± 3,500	340	21,000	4,400
PNC_300–1,000_ (1/cm^3^)	818	870 ± 710	28	4,800	830
PSC_total_ (μm^2^/cm^3^)	818	1,300 ± 800	78	5,100	960
PSC_50–100_ (μm^2^/cm^3^)	818	110 ± 49	10	330	60
PSC_100–300_ (μm^2^/cm^3^)	818	580 ± 350	29	2,100	440
PSC_300–1,000_ (μm^2^/cm^3^)	818	510 ± 430	17	2,700	490

Abbreviations: PNC_x_, particle number concentration in the given (x nm) or total size range (3 nm–1 μm); PSC_x_, particle surface area concentration in the given (x nm) or total size range (3 nm–1 μm).

**Table 2:** Correlation coefficients for daily mean values of meteorologic and air pollution variables.

Particle fraction	Air temperature (°C)	Relative humidity (%)	Air pressure (hPa)	SO_2_ (μg/m^3^)	NO_2_ (μg/m^3^)	PM_10_ (μg/m^3^)
UFP (1/cm^3^)	−0.22	−0.39	0.18	−0.26	0.06	0.05
PNC_total_ (1/cm^3^)	−0.25	−0.21	0.18	−0.18	0.27	0.23
PNC_3–10_ (1/cm^3^)	−0.22	−0.51	0.19	−0.19	−0.16	−0.09
PNC_10–30_ (1/cm^3^)	−0.02	−0.40	0.02	−0.32	−0.09	0.01
PNC_30–50_ (1/cm^3^)	−0.17	−0.15	0.16	−0.23	0.22	0.12
PNC_50–100_ (1/cm^3^)	−0.26	0.08	0.19	−0.03	0.43	0.23
PNC_100–300_ (1/cm^3^)	−0.13	0.36	0.05	0.15	0.55	0.44
PNC_300–1,000_ (1/cm^3^)	−0.02	0.47	−0.04	0.25	0.56	0.55
PSC_total_ (μm^2^/cm^3^)	−0.06	0.44	−0.02	0.23	0.58	0.55
PSC_50–100_ (μm^2^/cm^3^)	−0.26	0.12	0.18	−0.01	0.45	0.24
PSC_100–300_ (μm^2^/cm^3^)	−0.10	0.39	0.02	0.18	0.55	0.47
PSC_300–1,000_ (μm^2^/cm^3^)	−0.01	0.48	−0.05	0.25	0.56	0.56

**Table 3:** Overview of RR (95% CIs) for respiratory ERV per IQR increment of air pollutant in single-pollutant models.^a^

Pollutant	Time delay	IQR^b^	Cumulative effects model^c^ RR (95% CI)	PDL model^d^ RR (95% CI)	Single lag model^e^ (95% CI)
SO_2_	Same day	100	1.01 (0.97–1.05)	1.00 (0.96–1.04)	1.01 (0.97–1.05)
	5	100	1.04 (0.97–1.12)	1.01 (0.98–1.04)	1.01 (0.98–1.05)

NO_2_	Same day	40	1.02 (0.98–1.05)	1.01 (0.97–1.05)	1.02 (0.98–1.05)
	5	40	1.06 (1.00–1.12)*	1.01 (0.98–1.04)	1.02 (0.98–1.05)

PM_10_	Same day	90	1.01 (0.98–1.05)	1.01 (0.97–1.05)	1.01 (0.98–1.05)
	5	90	1.00 (0.94–1.06)	1.02 (0.99–1.06)	1.02 (0.99–1.05)

UFP	Same day	11,000	1.01 (0.95–1.07)	1.00 (0.94–1.07)	1.01 (0.95–1.07)
	5	11,000	0.99 (0.89–1.09)	0.93 (0.88–0.99)*	0.93 (0.88–0.97)*

PNC_total_	Same day	12,600	1.03 (0.98–1.09)	1.01 (0.95–1.08)	1.03 (0.98–1.09)
	1	12,600	1.04 (0.97–1.12)	1.06 (1.01–1.11)*	1.02 (0.96–1.08)
	2	12,600	1.07 (0.99–1.16)	1.04 (1.00–1.08)	1.03 (0.98–1.10)
	3	12,600	1.05 (0.96–1.15)	0.99 (0.95–1.03)	0.96 (0.91–1.02)
	4	12,600	1.04 (0.94–1.14)	0.96 (0.92–1.00)*	0.95 (0.90–1.00)
	5	12,600	1.03 (0.93–1.15)	0.97 (0.92–1.03)	0.94 (0.89–0.99)*

PNC_3–10_	Same day	3,880	0.97 (0.93–1.01)	0.98 (0.93–1.03)	0.97 (0.93–1.01)
	5	3,880	0.94 (0.86–1.02)	0.96 (0.92–0.99)*	0.96 (0.93–0.99)*

PNC_10–30_	Same day	4,300	0.98 (0.93–1.04)	0.99 (0.93–1.06)	0.98 (0.93–1.04)
	5	4,300	0.98 (0.88–1.10)	0.95 (0.90–1.00)*	0.95 (0.90–0.99)*

PNC_30–50_	Same day	2,300	1.03 (0.99–1.08)	1.02 (0.98–1.07)	1.03 (0.99–1.08)
	1	2,300	1.03 (0.97–1.09)	1.04 (1.00–1.07)*	1.00 (0.95–1.04)
	2	2,300	1.05 (0.98–1.12)	1.02 (0.99–1.05)	1.01 (0.96–1.05)
	3	2,300	1.04 (0.96–1.12)	0.99 (0.96–1.02)	0.98 (0.93–1.02)
	4	2,300	1.03 (0.94–1.12)	0.97 (0.94–1.00)	0.95 (0.91–0.99)*
	5	2,300	1.01 (0.92–1.11)	0.96 (0.92–1.01)	0.96 (0.91–1.00)

PNC_50–100_	Same day	3,600	1.03 (0.99–1.07)	1.01 (0.97–1.06)	1.03 (0.99–1.07)
	1	3,600	1.03 (0.98–1.09)	1.05 (1.01–1.08)*	1.00 (0.96–1.04)
	2	3,600	1.07 (1.00–1.15)*	1.03 (1.00–1.06)*	1.03 (0.98–1.07)
	3	3,600	1.08 (1.00–1.17)*	0.99 (0.97–1.02)	1.00 (0.96–1.04)
	4	3,600	1.05 (0.96–1.14)	0.98 (0.95–1.01)	0.96 (0.92–1.00)*
	5	3,600	1.06 (0.97–1.17)	1.01 (0.97–1.05)	0.99 (0.95–1.03)

PNC_100–300_	Same day	4,400	1.04 (1.00–1.08)	1.04 (0.99–1.08)	1.04 (1.00–1.08)
	1	4,400	1.05 (0.99–1.11)	1.05 (1.02–1.08)*	1.01 (0.97–1.06)
	2	4,400	1.09 (1.02–1.16)*	1.02 (0.99–1.04)	1.03 (0.99–1.07)
	3	4,400	1.08 (1.00–1.17)*	0.99 (0.97–1.01)	0.99 (0.96–1.03)
	4	4,400	1.05 (0.96–1.14)	0.98 (0.96–1.01)	0.98 (0.94–1.01)
	5	4,400	1.08 (0.99–1.18)	1.04 (1.00–1.08)*	1.02 (0.98–1.05)

PNC_300–1,000_	Same day	830	1.04 (1.00–1.08)	1.04 (0.99–1.08)	1.04 (1.00–1.08)
	5	830	1.03 (0.96–1.10)	1.04 (1.00–1.07)*	1.02 (0.99–1.05)

PSC_total_	Same day	960	1.05 (1.00–1.09)*	1.04 (1.00–1.08)	1.05 (1.00–1.09)*
	1	960	1.04 (0.99–1.10)	1.03 (1.00–1.05)*	1.01 (0.98–1.05)
	2	960	1.05 (0.99–1.11)	1.00 (0.98–1.02)	1.01 (0.98–1.05)
	3	960	1.04 (0.98–1.11)	0.98 (0.96–1.00)	0.99 (0.96–1.03)
	4	960	1.02 (0.95–1.09)	0.99 (0.97–1.01)	0.99 (0.96–1.02)
	5	960	1.04 (0.97–1.12)	1.04 (1.01–1.08)*	1.02 (0.99–1.06)

PSC_50–100_	Same day	60	1.03 (0.99–1.07)	1.01 (0.97–1.06)	1.03 (0.99–1.07)
	1	60	1.03 (0.98–1.09)	1.05 (1.02–1.08)*	1.00 (0.96–1.04)
	2	60	1.07 (1.01–1.15)*	1.03 (1.00–1.06)*	1.03 (0.99–1.07)
	3	60	1.09 (1.01–1.17)*	1.00 (0.97–1.02)	1.00 (0.96–1.04)
	4	60	1.05 (0.96–1.14)	0.98 (0.95–1.01)	0.96 (0.93–1.00)
	5	60	1.07 (0.97–1.17)	1.01 (0.97–1.05)	0.99 (0.95–1.03)

PSC_100–300_	Same day	440	1.04 (1.00–1.09)*	1.04 (1.00–1.09)	1.04 (1.00–1.09)*
	1	440	1.05 (0.99–1.11)	1.04 (1.01–1.07)*	1.02 (0.98–1.06)
	2	440	1.08 (1.01–1.15)*	1.01 (0.99–1.04)	1.02 (0.99–1.06)
	3	440	1.07 (1.00–1.15)	0.99 (0.96–1.01)	0.99 (0.95–1.03)
	4	440	1.04 (0.96–1.13)	0.99 (0.96–1.01)	0.98 (0.95–1.02)
	5	440	1.07 (0.98–1.17)	1.04 (1.00–1.08)*	1.02 (0.98–1.06)

PSC_300–1,000_	Same day	490	1.04 (1.00–1.08)	1.04 (0.99–1.08)	1.04 (1.00–1.08)
	1	490	1.03 (0.99–1.08)	1.01 (0.99–1.04)	1.01 (0.98–1.05)
	5	490	1.02 (0.96–1.09)	1.04 (1.01–1.07)*	1.02 (0.99–1.06)

a For a complete table, see Supplemental Material, Table 2 (doi:10.1289/ehp.1002203).

b Units for IQR: SO_2_, NO_2_, and PM_10_ (μg/m^3^); PNC_x_ and UFP (1/cm^3^); PSC_x_ (μm^2^/cm^3^).

c Cumulative-effects models represent time-delayed effects with moving averages up to 6 days (mean of the same day and 5 previous days).

d PDL models with the lag coefficients follow a third-degree polynomial of the lag number and a maximum lag of 5 days.

e Single lag models represent time-delayed effects with lagged effect up to 5 days.

* *p* < 0.05 (*p*-values for the null hypothesis that the corresponding parameter is zero).

**Table 4:** Overview of RRs (95% CIs) between respiratory ERV and an IQR increment of air pollutant while controlling for NO_2_ or PM_10_.^a^

Pollutant	Time delay (days)	IQR^b^	While controlling for NO_2_	While controlling for PM_10_
NO_2_	3	40	—	1.07 (1.01–1.13)*
	4	40	—	1.07 (1.01–1.14)*
	5	40	—	1.08 (1.01–1.15)*

PNC_50–100_	2	3,600	1.06 (0.99–1.14)	1.07 (1.00–1.15)*
	3	3,600	1.06 (0.98–1.16)	1.08 (1.00–1.17)*

PNC_100–300_	2	4,400	1.08 (1.00–1.17)	1.10 (1.02–1.19)*
	3	4,400	1.06 (0.97–1.16)	1.11 (1.02–1.21)*

PSC_50–100_	2	60	1.06 (0.99–1.14)	1.07 (1.01–1.15)*
	3	60	1.07 (0.98–1.16)	1.09 (1.01–1.17)*

PSC_100–300_	2	440	1.07 (0.99–1.16)	1.10 (1.02–1.19)*
	3	440	1.05 (0.95–1.15)	1.10 (1.01–1.20)*

a For a complete table, see Supplemental Material, Table 3 (doi:10.1289/ehp.1002203). Estimates were calculated using cumulative effects models representing time-delayed effects with moving averages up to 6 days (mean of the same day and 5 previous days) and including both pollutants with the same lag, for example, same-day total particle number concentration and same-day NO_2_ concentration.

b Units for IQR: NO_2_ (μg/m^3^); PNC_x_ (1/cm^3^); PSC_x_ (μm^2^/cm^3^).

* *p* < 0.05 (*p*-values for the null hypothesis that the corresponding parameter is zero).
